# Adenomyosis: An Updated Review on Diagnosis and Classification

**DOI:** 10.3390/jcm12144828

**Published:** 2023-07-21

**Authors:** Gaby Moawad, Arrigo Fruscalzo, Youssef Youssef, Mira Kheil, Tala Tawil, Jimmy Nehme, Paul Pirtea, Benedetta Guani, Huda Afaneh, Jean Marc Ayoubi, Anis Feki

**Affiliations:** 1 Department of Obstetrics and Gynaecology, The George Washington University Hospital, Washington, DC 20037, USA; 2 Department of Obstetrics and Gynecology, HFR—Hòpital Fribourgeois, Chemin des Pensionnats 2-6, 1708 Fribourg, Switzerland; 3 Division of Minimally Invasive Gynecologic Surgery, Department of Obstetrics and Gynaecology—Maimonides Medical Center, Brooklyn, NY 11219, USA; 4 Department of Obstetrics & Gynecology, Henry Ford Health, Detroit, MI 48202, USA; 5 Department of Pathology, Henry Ford Health, Detroit, MI 48202, USA; 6 Department of Internal Medicine, Henry Ford Health, Detroit, MI 48202, USA; 7 Department of Obstetrics and Gynaecology and Reproductive Medicine, Hopital Foch-Faculté de Médecine Paris, 92150 Suresnes, France; 8 Department of Obstetrics and Gynecology, Women’s Health Institute, Cleveland Clinic Foundation, Cleveland, OH 44195, USA; afanehhu@gmail.com

**Keywords:** adenomyosis, abnormal uterine bleeding, dysmenorrhea, imaging, histopathology, junctional zone, MRI, pelvic pain, ultrasound, uterine disorders

## Abstract

Adenomyosis is a commonly diagnosed benign condition characterized by the presence of ectopic endometrial glands within the underlying myometrium. The most common presenting signs and symptoms are abnormal uterine bleeding, chronic pelvic pain, and infertility. The clinical relevance of this condition is evident in both medical and surgical care. Histopathology and imaging studies are used for the diagnosis and classification of adenomyosis, which are hallmarks of the advancement of our ability to diagnose adenomyosis. Importantly, the diagnosis and classification of adenomyosis lacks standardization due to the nature of imaging techniques, features of adenomyosis, and the clinical spectrum of adenomyosis. We reviewed the literature to summarize the available classification systems for adenomyosis and highlight the different imaging approaches and histologic criteria used in diagnosis. Despite the high prevalence of the disease, there is no clear consensus on one classification system. We provide a review of some of the classification systems available and discuss their strengths and limitations.

## 1. Introduction

Adenomyosis is a benign condition of the uterus, historically diagnosed based on histology after hysterectomy; specifically by visualizing ectopic endometrial glands and stroma at a minimum depth of 2.5 mm below the endomyometrial junction with a hypertrophic and hyperplastic surrounding myometrium [1,2]. As imaging techniques further developed, a non-invasive approach to diagnosing adenomyosis became feasible, allowing for the earlier clinical detection of the disease. Magnetic resonance imaging (MRI) and transvaginal ultrasonography (TVUS) are reported to have similar sensitivities and specificities in the detection of adenomyosis, and have increasingly been used to identify the presence of adenomyotic lesions, and to plan subsequent treatment [3].

Adenomyosis has been described as both diffuse and focal, depending on its distribution within the myometrium. Diffuse adenomyosis is defined by the presence of multiple foci within the uterine myometrium, while focal adenomyosis appears as isolated nodules of hypertrophic myometrium and ectopic endometrium [4,5]. The pathogenesis of adenomyosis remains vaguely understood, however, and the relationship between the extent of disease and clinical manifestation is still unclear, making standardized treatment difficult to determine.

Attempts have been made to classify adenomyosis into subtypes according to the findings of histopathology and imaging, but none of the proposed systems have been adopted into practice [5,6,7,8,9]. This is essentially because these systems have not shown adequate clinical correlations to allow therapeutic guidance. There is a growing need for a validated classification system that correlates with the clinical severity of the disease, especially with the development of new therapeutic options for adenomyosis. Such a system would lay out a reliable framework allowing researchers to compare data and clinicians to evaluate symptoms and tailor therapeutic plans. We aim to review the current proposed classification systems for adenomyosis and highlight their strengths and limitations. Relating each of these classification systems to clinical practice may provide an opportunity to optimize treatment for adenomyosis.

## 2. Materials and Methods

A literature search was performed on PubMed using the keywords “adenomyosis AND classification”, “adenomyosis AND histology”, “adenomyosis AND imaging”, “adenomyosis AND ultrasonography OR ultrasound”, “adenomyosis AND magnetic resonance imaging”. The search was limited to the English language and to full-text articles. Reviews, case reports, and commentaries were excluded. Abstracts were screened by two authors, and relevant articles were selected.

## 3. Results

### 3.1. Retrieved Articles

**Figure 1:**
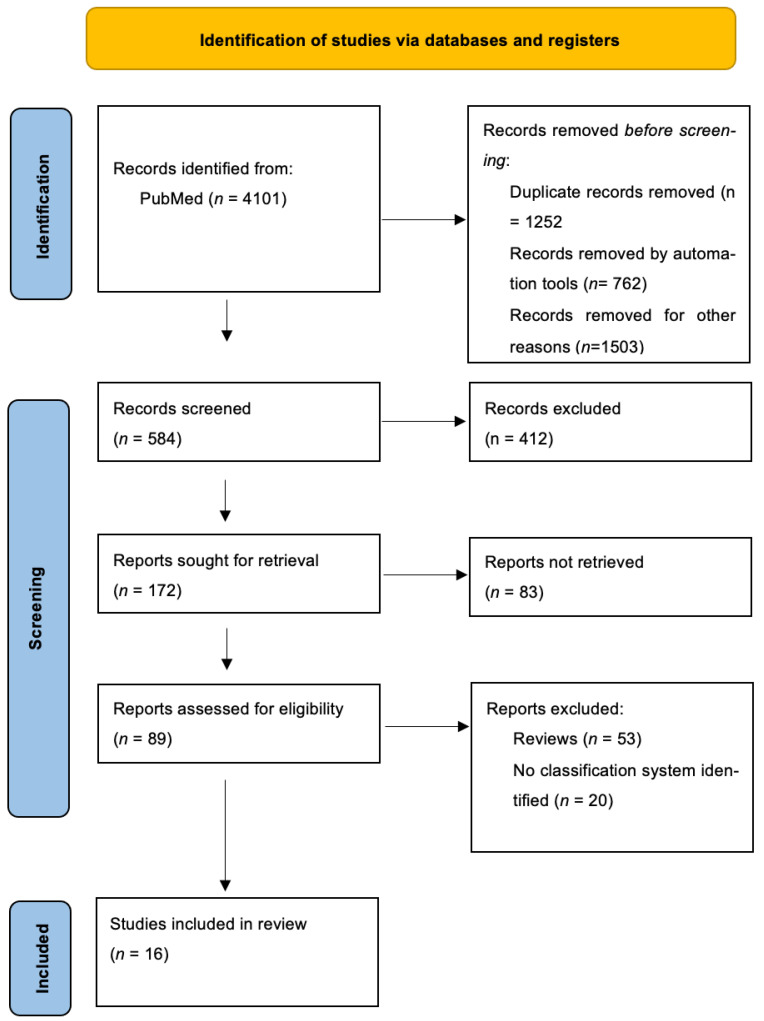
Flow diagram of study identification and selection.

We ran five searches on PubMed, which yielded a total of 4101 articles. This number decreased to 3339 after limiting results to the English language and to full text articles. Duplicates and irrelevant records were removed. A total of 584 abstracts were screened. After excluding reviews, commentaries, case reports, and articles in which no classification system was identified, we included 16 articles for the purposes of this review (Figure 1).

#### 3.1.1. Current Classification Systems

Various categories of adenomyosis have been described based on histologic, sonographic, and magnetic resonance imaging features, and emerging classification systems have thus been either histopathology-based or imaging-based. We will outline some of the various criteria developed in both of these categories below.

##### Histopathology

To date, there is no universally accepted histological classification system for adenomyosis. A correlation between response to treatment and histological findings of adenomyosis has been demonstrated across several studies, highlighting the importance of having a standard classification system for guiding treatment [1,2,10,11,12]. Several studies have further investigated the correlation between severity of clinical symptoms and histopathologic features, mainly the depth of penetration and degree of spread, assessed by the number of foci and number of glands [1,11,13].

Bird et al. first classified adenomyotic lesions according to the depth of penetration, reflected in the uterine layer affected; and the extent of involvement, measured by the number of endometrial glands seen per low-power field (Table 1) [1]. According to Bird et al., Grade I represent adenomyosis sub-basalis/sub-endometrial basalis (adenomyosis within one low-power field below the “basal” endometrium, but with no further penetration); Grade II represents adenomyosis penetration to the mid-myometrium; and Grade III represents adenomyosis penetration beyond the mid-myometrium. The authors further demonstrated a direct correlation between the severity of dysmenorrhea and the depth of penetration, such that 4.3% of women with Grade I adenomyosis reported experiencing dysmenorrhea, compared to 42.4% with Grade II and 83.3% with Grade III [1].

Another histopathologic feature that has been described and is seen mainly in those with deep penetration is hemosiderin deposition adjacent to adenomyotic lesions caused by bleeding of the ectopic endometrium. This suggests that hemosiderin deposition may reflect the extent and severity of adenomyosis; however, the significance of this finding remains unclear [1,2].

Several other systems follow similar criteria for categorizing adenomyosis. Nishida et al. described two types of adenomyosis depending on the location of the lesions. Type I involves adenomyosis that is continuous from the surface endometrium, and Type II involves adenomyosis present on the serosa of the uterus and not continuous from the surface endometrium. They graded the degree of involvement according to number of glands and stroma (islands of adenomyotic lesions) [11]. Nishida et al. further evaluated the severity of dysmenorrhea based on various histologic features of adenomyosis, and found significant differences when assessing the number of islands of adenomyosis, number of glands, distance between the surface endometrium, deepest adenomyotic lesion, and the ratio of the depth of adenomyosis to uterine muscle thickness [11]. On the other hand, there were no significant differences when assessing muscle layer thickness, number of glands per island of adenomyosis, or the presence or absence of hemorrhage in adenomyosis [11]. Dysmenorrhea was noted when glandular invasion exceeded 80% or more of the myometrium, but there was no clear cut-off regarding the number of islands, glands, or depth of adenomyosis [11]. The authors also demonstrated an association between CA 125 levels and the severity of dysmenorrhea [11].

Other classification systems are based on the depth of myometrial involvement, and divide adenomyosis into either two or three grades [10,12,14,15,16]. Levgur et al. described the depth of adenomyosis as a percentage of myometrial thickness such that superficial is less than 40% of myometrial thickness; intermediate is 40–80% of thickness; and deep exceeds 80% of thickness. They further noted the presence of dysmenorrhea in 77.8% of patients with deep myometrial foci, compared to 12.5% with intermediate foci. Superficial myometrial foci were not associated with dysmenorrhea or menorrhagia [12].

As in older classifications, a few of the newer ones use the number of adenomyotic foci or islets to categorize the severity of adenomyosis [10,12,16]. According to Vercenilli et al., Grade 1 corresponded to 1–3 islets, Grade 2 to 4–10 islets, and Grade 3 to more than 10 islets.

In addition to using the percentage of myometrial penetration, Sammour et al. classified the degree of spread of adenomyosis by the number of foci and the extent of disease according to a “penetration ratio”, described as the depth of penetration to myometrial thickness [2]. The authors found a direct correlation between the spread of adenomyosis and dysmenorrhea, but not the depth of penetration. Interestingly, studies have shown that the degree of spread (number of adenomyosis foci) and depth of penetration are positively correlated [11,12]. Sammour et al.’s findings are thus still in line with previous studies [1,11,12]. The association between dysmenorrhea and severity of disease could possibly be attributed to coexisting conditions which are commonly present with adenomyosis, such as leiomyomas and endometriosis [1,17,18]. However, Levgur et al. described patients with coexisting conditions and demonstrated that the correlation between dysmenorrhea and severity of disease is still significant regardless of confounding factors [12].

Hulka et al. later introduced a new category for focal adenomyosis in which they coined the term “adenomyoma”, in addition to the two other categories that distinguished disease involving the inner versus outer myometrium [15].

Most recently, Rassmussen et al. introduced a histological classification based on endomyometrial biopsies from transcervical endometrial resection (TCRE). They defined adenomyosis as a disease of the junctional zone with ectopic endometrium infiltrating the myometrium and required a thick biopsy with a depth of ≥5 mm into the myometrium to obtain an accurate assessment of the junctional zone. They classified adenomyosis into intrinsic, serrated JZ, and linear JZ [13]. Intrinsic adenomyosis involves ≥2 mm myometrial invasion without contact with the basal endometrium; serrated junctional zone represents >3 mm myometrial invasion with contact with the basal endometrium (precursor of adenomyosis); and linear junctional zone corresponds to no or marginal myometrial invasion ≤3 mm with contact with the basal endometrium [16].

Rasmussen et al. investigated the relationship between the response to transcervical endometrial resection and the degree of junctional zone changes. Reintervention surgery was more commonly required in women with intrinsic adenomyosis compared to women with either linear or serrated junctional zone adenomyosis [13]. At six months follow-up, patients with linear junctional zone had more symptom relief compared to patients with intrinsic adenomyosis or serrated junctional zone adenomyosis [13].

**Table 1:** Histopathologic classifications of adenomyosis.

Author, Year	Name	Depth	Foci
Bird et al., 1972 [1]	Grade I—Slight	Sub-endometrial basalis	1–3 foci/low power field (LPF)
	Grade II—Moderate	Mid-myometrium	4–9
	Grade III—Marked	Outer myometrium	≥10
Nishida et al., 1991 [11]	Type I	Continuous from the surface endometrium	Islands/section
	Type II	Continuous from serosa	Glands/section
McCausland, 1992 [14]	Superficial	≤1 mm depth	-
	Deep	>1 mm depth	-
Siegler et al., 1994 [16]	Grade 1—Mild	Inner 1/3	1–3 foci/LPF
	Grade 2—Moderate	2/3	4–9
	Grade 3—Severe	Entire myometrium	>10
Levgur et al., 2000 [12]	Superficial	<40%	Foci/LPF
	Intermediate	40–80%	-
	Deep	>80%	-
Sammour et al., 2002 [2]	-	<25%	Foci/slide
	-	26–50%	
	-	51–75%	-
	-	>75%	-
Hulka et al., 2002 [15]	Category 1: Mild	Inner 1/3 (or microscopic foci)	-
	Category 2: Focal	Adenomyoma	-
	Category 3: Severe/diffuse	Outer 2/3 (include entire myometrium)	-
Vercellini et al., 2006 [10]	Mild—Grade 1	Up to 1/3	1–3 islets
	Moderate—Grade 2	1/3 to 2/3	4–10 islets
	Severe—Grade 3	>2/3	>10 islets
Rasmussen et al., 2019 [13]	Intrinsic	≥2 mm myometrial invasion without contact with the basal endometrium	-
	Serrated junctional zone	>3 mm myometrial invasion with contact with the basal endometrium (precursor of adenomyosis)	-
	Linear junctional zone	No or marginal myometrial invasion ≤3 mm with contact with the basal endometrium	

###### Ultrasonography

In 2015, the Morphological Uterus Sonographic Assessment (MUSA) group released a consensus statement that included a description of the features typically seen on TVUS in patients with adenomyosis. These included an enlarged globular uterus, asymmetrical thickening of the myometrium, myometrial cysts, echogenic subendometrial lines and buds, hyperechogenic islands, fan-shaped shadowing, an irregular or interrupted junctional zone, and translesional vascularity on color Doppler ultrasound examination [19]. The MUSA criteria were revised in 2021, and further subdivided into direct and indirect features [20]. Even though the MUSA criteria provided uniform guidance for recognizing and identifying adenomyotic lesions, it did not establish a classification system for the disease. TVUS is easily available and cost effective, and so classification systems that rely on sonographic findings are encouraged. Two such systems have been proposed to date.

Lazzeri et al. conducted a study in 2018 during which two gynecologists independently scored the ultrasounds of patients with adenomyosis according to a scoring system based on the type (diffuse vs. focal vs. adenomyoma), location in the myometrium (outer vs. inner/junctional zone), number of adenomyotic lesions, their size, and the extent of myometrial involvement (Table 2) [9]. A comparison of interobserver variability showed near perfect agreement. This system was later shown to have clinical relevance in a follow-up study. The investigators classified 108 patients with sonographic signs of adenomyosis according to the proposed system, and found that patients with diffuse disease were older and had heavier menstrual bleeding compared to those with focal adenomyosis, while those with focal disease tended to have higher rates of infertility [21]. Focal disease that involved the junctional zone was also correlated with a higher proportion of miscarriages compared to diffuse disease. It is worth noting, however, that this system did not correlate with the presence or severity of dyspareunia and dysmenorrhea, thus maybe lacking clinical correlation [21].

In 2019, Bosch et al. [5] suggested another TVUS classification system for adenomyosis, comprised of the following components:

(a) Presence of adenomyosis: assessed by the MUSA criteria;
(b) Location of lesions in the uterus: anterior, posterior, lateral left, lateral right or fundal;
(c) Type of adenomyosis: termed focal when >25% of the lesion circumference is surrounded by normal myometrium provided that <25% of the entire myometrium is involved; and diffuse with involvement of >25% of the myometrium. When a focal lesion is well demarcated and surrounded by hypertrophic myometrium, it is an adenomyoma;
(d) Presence or absence of cysts;
(e) Myometrial layer involvement: Type 1 involves the junctional zone (JZ), also termed inner myometrium, Type 2 involves the middle myometrium, Type 3 involves the outer myometrium (subserosal);
(f) Extent of uterine involvement: estimated subjectively and reported as mild <25%, moderate 25–50%, and severe >50%;
(g) Lesion size: through measuring the largest diameter of focal lesions or myometrial wall thickness involvement of diffuse adenomyosis.

This system was not externally validated, however, and its correlation to clinical symptoms has not been studied. The feasibility and relevance of differentiating between the myometrial layers on ultrasound also needs to be further investigated [5].

###### MRI

The most important MRI finding for the diagnosis of adenomyosis is the presence of JZ thickening >12 mm [22]. Another significant finding is high-intensity myometrial foci on T2 or T1 weighted images [4,23]. A wide range of sensitivities and specificities have been reported when using MRI in diagnosing adenomyosis. A pooled analysis of studies showed a sensitivity of around 78% and specificity of 93% [23]. Although TVUS is also reported to have similar sensitivity and specificity to MRI, studies assessing the diagnostic accuracy of TVUS are too heterogenic to be pooled [24]. This is probably due to the operator-dependent subjectivity in ultrasonography. MRI-based systems thus provide more objectivity and consistency in classifying adenomyosis. MRI imaging can distinguish uterine zonal anatomy and visualize the JZ, allowing lesions to be located to the central endometrium or outer myometrium.

Gordts et al. described three separate entities based on imaging analysis of the uterine JZ in the MRI of patients with adenomyosis: JZ hyperplasia involving only the JZ, adenomyosis extending into the myometrium, and adenomyoma described as a mass with indistinct margins [6]. Grimbizis et al. based their classification on the extent of myometrial invasion seen on imaging, and added a histologic component [25]. The main subgroups they described were diffuse, focal, polypoid, and others. They further classified focal into adenomyoma and cystic adenomyosis; polypoid into typical and atypical depending on the presence or absence of cellular atypia; and others including endocervical and retroperitoneal.

Kishi et al. described four subtypes of adenomyosis based on the geographic interrelationship between the lesions and other structural components of the uterus (Table 3). The authors hypothesized that the subtypes could constitute different pathologies that present clinically with similar symptoms [7]. Their analysis showed that uterorectal adhesions, posterior cul-de-sac obliteration and posterior cul-de-sac endometriosis were associated with Subtype II but not Subtype I, while anterior wall involvement and history of curettage were associated with Subtype I but not Subtype II. This inverse relationship could imply the presence of distinct pathologies in the development of each subtype. Subtype I is thought to be due to direct endometrial invasion into the myometrium, and Subtype II is hypothesized to be a successor of endometriotic invasion, given the high rate of concurrent endometriosis seen. The authors suggested that Subtype III arises from de novo metaplasia, while Subtype IV represents an advanced form of any, or multiple of the other types, and has no clear distinction from the normal uterus [7]. There was a significantly high frequency and severity of menorrhagia and dysmenorrhea in all patients with Subtype IV, and thus Kishi et al. concluded that these symptoms correlate with the extent and depth of adenomyosis [7].

Bazot et al. proposed a three-tier classification system and divided adenomyosis into internal, external, and distinct adenomyomas [24]. Their system also introduced distinctions based on the location and components of the lesions (Table 3). They suggested that their system is potentially related to therapeutic strategy, but this was not clinically evaluated.

Several authors have also described systems that combine criteria from previously proposed classifications [26,27]. One of the most comprehensive and recent of these is the classification system suggested by Kobayashi et al. [8], which encompasses five components and grades them as follows:

- Affected area:
  A: internal adenomyosis (inner myometrium);
  B: external adenomyosis (outer myometrium);
- Pattern: diffuse vs. focal;
- Size: reported as 1 (<1/3), 2 (<2/3), or 3 (>2/3 of the uterine wall);
- Concomitant pathologies: none, peritoneal endometriosis, ovarian endometrioma, deep infiltrating endometriosis, uterine fibroids, others; reported as C_0–5_, respectively;
- Localization: anterior, posterior, left lateral, right lateral, fundus; reported as D_1–5_, respectively;
- The final score is then reported as the four letters with their corresponding numbers according to MRI findings (Table 3).

**Table 3:** Summary of MRI-based classification systems of adenomyosis.

Author, Year	Classification		Criteria
Gordts et al., 2008 [6]	JZ hyperplasia		JZ thickness measuring ≥8 mm but <12 mm on T2-weighted images in women aged 35 years or less. Partial or diffuse type
	Adenomyosis		JZ thickness ≥12 mm; high-signal intensity myometrial foci; involvement of the outer myometrium: <1/3, <2/3, >2/3
	Adenomyoma		Myometrial mass with indistinct margins of primarily low-signal intensity on all MR sequences. Retrocervical, retrovaginal, fallopian tube, and bladder types
Kishi et al., 2012 [7]	Subtype I (intrinsic)		Adenomyosis has an intimate relationship with inner structural components of the uterus such as the endometrium and JZ
	Subtype II (extrinsic)		Adenomyosis arising from the outer shell of the uterus, disrupting the serosa but not affecting the inner components
	Subtype III (intramural)		Adenomyosis residing solely in the myometrium
	Subtype IV (others)		Indeterminate, does not fit into other subtypes
Grimbizis et al., 2014 [25]	Diffuse		Foci of endometrial mucosa scattered throughout the uterine musculature
	Focal	Adenomyoma	Infiltration of a restricted area of the myometrium with clear borders and mainly solid characteristics
		Cystic adenomyosis	Single adenomyotic cyst within the myometrium
	Polypoid	Typical	Circumscribed endometrial masses composed of endometrioid glands and smooth muscle without architectural or cellular atypia
		Atypical	A variant of polypoid with atypical endometrial glands and cellular smooth muscle stroma
	Other	Endocervical	Adenomyomatous polyps in the cervix that contain epithelial component of endocervical type
		Retroperitoneal	Nodules thought to arise from metaplasia of Müllerian remnants beneath the peritoneum and in the upper rectovaginal septum
Dashottar et al., 2015 [26]	Focal		Focal widening of the JZ ≥14 mm
	Diffuse	Even	Consistent JZ thickening ≥14 mm throughout the uterus
		Uneven	Variable JZ thickening ≥14 mm throughout the uterus
Bazot et al., 2018 [24]	Internal adenomyosis	Focal	Localized intramyometrial tiny cystic component with or without JZ bulging
		Superficial	Disseminated subendometrial tiny cystic component without JZ hypertrophy
		Diffuse	Disseminated intramyometrial tiny cystic component with JZ hypertrophy
	Adenomyomas	Intramural solid	Ill-defined myometrial lesion with tiny cystic component
		Intramural cystic	Ill-defined myometrial lesion with hemorrhagic cystic cavity
		Submucosal	Ill-defined myometrial lesion with tiny cystic component and intracavitary protrusion
		Subserosal	Ill-defined subserous myometrial lesion with tiny cystic component
	External adenomyosis	Posterior	Ill-defined posterior myometrial mass associated with posterior deep endometriosis
		Anterior	Ill-defined subserosal anterior myometrial mass associated with anterior deep endometriosis
Kobayashi et al., 2020 [24]	Affected area	A	Internal adenomyosis, thickness of JZ >12 mm
		B	External adenomyosis, thickness of JZ <8 mm
	Size & pattern	A_1_ or B_1_	<1/3 of uterine wall, mostly focal
		A_2_ or B_2_	<2/3 of uterine wall, can be focal or diffuse
		A_3_ or B_3_	>2/3 of uterine wall, mostly diffuse
	Concomitant pathologies	C_0–5_	None C_0_, peritoneal endometriosis C_1_, ovarian endometrioma C_2_, deep infiltrating endometriosis C_3_, uterine fibroids C_4_, others C_5_
	Location	D_1–5_	Anterior D_1_, posterior D_2_, left lateral D_3_, right lateral D_4_, fundus D_5_

## 4. Discussion

Adenomyosis is a common gynecological condition characterized by the presence of endometrial tissue within the myometrium, which can lead to debilitating symptoms such as dysmenorrhea, menorrhagia, and infertility. Diagnosis of adenomyosis can be challenging as it requires a combination of clinical evaluation, imaging, and histopathological examination. Several classification systems have been proposed to categorize adenomyosis based on different features such as the depth of invasion, the extent of involvement, and the location of lesions, none of which have been adopted clinically. Our review summarizes different imaging and histologic criteria, with several strengths and areas of future work.

Proposed systems are based on findings seen either on histopathology or imaging, which help determine the presence or absence of adenomyosis and its anatomical location. A distinction needs to be made between categorizing the disease based on visualizing certain characteristics, however, versus rooting the classification in symptom severity and prognostic indicators. Similar to endometriosis and fibroids, the topography of adenomyosis is unlikely to correlate to clinical symptoms which limits the clinical utility of the classification systems.

As seen in other types of medical entities, classifications are needed to predict specific outcomes of interest. There are essentially three issues to be addressed when developing and evaluating a classification system for adenomyosis: (1) Does classification correlate with the severity of symptoms? (2) Does classification correlate with fertility and with outcomes of reproductive medicine? (3) Does classification correlate with the indication and type of treatment and with its success? These questions can be applied towards the various classification systems in an attempt to narrow down criteria that are associated with clinical outcomes.

To correctly classify a pathology, a feasible, reliable, and accurate diagnostic test is required. Each proposed classification model should thus be evaluated and compared according to these hallmarks. Much like many tests, the least invasive is often preferred for patient care. Developing and testing a classification system based on radiologic features is preferred over a histological classification, which can currently only be performed in a reliable manner after the hysterectomy. Habiba et al. discusses the challenges of standardizing histological criteria due the varying degrees of myometrial invasion and subjectivity among histopathologists [28]. Nonetheless, the efforts being made to develop a histological classification are valuable in understanding the pathophysiology of adenomyosis. The second and third pillars: reproducibility and diagnostic accuracy, have not yet been extensively evaluated for most classifications proposed, and should be evaluated concerning the chosen diagnostic test and outcomes.

In addition, imaging modalities such as MRI and Ultrasound can have a level of subjectivity that can make it difficult to standardize a classification system. In the case of MRI, the junctional zone is utilized to define adenomyosis, but there are several variables that influence the cut-off point measurement. These include age parity, menstrual cycle phase, surgical history and inability to visualize the JZ [28]. Transvaginal ultrasound does appear to have a high sensitivity and specificity of detecting adenomyosis (Celli and Habiba article), but a proportion of individuals with adenomyosis may still have a normal ultrasound and there are operator differences. MRI is the modality of choice in an inconclusive ultrasound, due to its ability to effectively differentiate tissue, which can also be helpful for focal adenomyosis and identifying endometriotic lesions [29].

It is necessary to discuss future applications of AI in medicine, specifically related to our topic of adenomyosis classification. Systems based on JZ abnormalities have shown promising results in terms of interobserver agreement and correlation with clinical symptoms [6,9]. Recently, there has been growing interest in the potential of artificial intelligence (AI) to improve the accuracy and consistency of adenomyosis diagnosis and classification. It has been suggested that AI-based analysis of US or MRI images could potentially accurately identify and classify different types of adenomyosis based on JZ abnormalities [30,31]. This approach has the potential to improve the standardization and reproducibility of adenomyosis diagnosis since AI algorithms can analyze large datasets and identify patterns that may not be immediately apparent to human clinicians. However, much like many areas of medicine, further research is needed to evaluate these approaches to explore whether they provide answers to the clinically relevant questions.

The strengths of our review include being able to identify in the literature specific studies that discuss the histologic and imaging classification systems. We provide a summary of the histologic and imaging criteria that aid in the diagnosis of adenomyosis with their common features that may be more reproducible in the future. Areas of future work include further evaluating whether the classification systems have correlated to more tailored treatment algorithms for individuals with adenomyosis symptoms. We also hope that further classification systems will continue to develop, as we found only 16 articles that matched our inclusion criteria. The increased research in the area of histology and imaging with clinical findings will also strengthen the available classification systems.

## 5. Conclusions

Despite progress in identifying unique features for the detection of adenomyosis through histology and imaging, clinical management and outcomes remain highly variable and inconsistent in practice. There have been attempts to classify adenomyosis into clinically relevant categories but none of the proposed systems has been adopted. This review brings to light the various approaches using histologic and imaging criteria. The first step is to identify the potential classification systems and their ability to correlate with clinical findings. The need for a classification system that allows clinicians to grade the disease and plan treatments accordingly remains unmet and must be prioritized for the purpose of improving the management of this condition and meeting patient treatment goals and improving their quality of life.

## Figures and Tables

**Table 2:** Lazzeri et al.’s sonographic mapping of adenomyosis [9].

Score	Diffuse Outer Myometrium	Diffuse Inner Myometrium/JZ	Focal Outer Myometrium	Focal Inner Myometrium/JZ	Adenomyoma
1	1 myometrial wall involvement with myometrial wall thickness ≤20 mm	maximum JZ thickness (JZ max) >6 to ≤8 mm difference (JZmax) − (JZmin) = JZdif. >4 to ≤6 mm diffuse infiltration of the JZ ≤20 mm in length	1 focal intramyometrial lesion ≤10 mm	1 focal lesion of the JZ by hyperechoic tissue or cystic areas ≤10 mm	1 adenomyoma with the largest diameter ≤20 mm
2	2 myometrial wall involvements with wall thickness ≤20 mm 1 myometrial wall involvement with wall thickness >20 to ≤30 mm	maximum JZ thickness (JZ max) >8 mm difference (JZmax) − (JZmin) = JZdif. >6 mm diffuse infiltration of the JZ <20 mm in length or <50% of the uterus	≥2 focal intramyometrial lesion ≤10 mm 1 focal intramyometrial lesion >10 to ≤20 mm	≥2 focal lesions of the JZ ≤10 mm 1 focal lesion of the JZ >10 to ≤20 mm	2 adenomyoma with the largest diameter ≤20 mm 1 adenomyoma with the largest diameter >20 to ≤30 mm
3	1 myometrial wall involvement with wall thickness >30 mm 2 myometrial wall involvements with wall thickness >20 to ≤30 mm	diffuse infiltration of the JZ >50% to ≤80% of the uterus	≥2 focal intramyometrial lesion >10 to ≤20 mm 1 focal intramyometrial lesion >20 mm	≥2 focal lesions of the JZ >10 to ≤20 mm 1 focal lesion of the JZ >20 mm	2 adenomyoma with the largest diameter >20 to ≤30 mm 1 adenomyoma with the largest diameter >30 to ≤40 mm
4	2 myometrial wall involvements with wall thickness >30 mm all uterus involvements with globally enlarged uterus	80% total infiltration of the JZ difference (JZmax) − (JZmin) = JZdif. >4 to ≤6 mm diffuse infiltration of the JZ ≤20 mm in length	≥2 focal intramyometrial lesion >20 mm	≥2 focal lesions of the JZ >20 mm	1 or more adenomyoma with the largest diameter >40 mm

## Data Availability

All data generated or analyzed during this study are included in this published article.
